# The burden and management of anemia in Greek patients with inflammatory bowel disease: a retrospective, multicenter, observational study

**DOI:** 10.1186/s12876-021-01826-1

**Published:** 2021-06-29

**Authors:** Kalliopi Foteinogiannopoulou, Konstantinos Karmiris, Georgios Axiaris, Magdalini Velegraki, Antonios Gklavas, Christina Kapizioni, Charalabos Karageorgos, Christina Kateri, Anastasia Katsoula, Georgios Kokkotis, Evgenia Koureta, Charikleia Lamouri, Panagiotis Markopoulos, Maria Palatianou, Ploutarchos Pastras, Konstantinos Fasoulas, Olga Giouleme, Evanthia Zampeli, Aggeliki Theodoropoulou, Georgios Theocharis, Konstantinos Thomopoulos, Pantelis Karatzas, Konstantinos H. Katsanos, Andreas Kapsoritakis, Anastasia Kourikou, Nikoleta Mathou, Spilios Manolakopoulos, Georgios Michalopoulos, Spyridon Michopoulos, Alexandros Boubonaris, Giorgos Bamias, Vasileios Papadopoulos, George Papatheodoridis, Ioannis Papaconstantinou, Ioannis Pachiadakis, Konstantinos Soufleris, Maria Tzouvala, Christos Triantos, Eftychia Tsironi, Dimitrios K. Christodoulou, Ioannis E. Koutroubakis, the Hellenic group for the study of IBD, Kalliopi Foteinogiannopoulou, Konstantinos Karmiris, Georgios Axiaris, Magdalini Velegraki, Antonios Gklavas, Christina Kapizioni, Charalabos Karageorgos, Christina Kateri, Anastasia Katsoula, Georgios Kokkotis, Evgenia Koureta, Charikleia Lamouri, Panagiotis Markopoulos, Maria Palatianou, Ploutarchos Pastras, Konstantinos Fasoulas, Olga Giouleme, Evanthia Zampeli, Aggeliki Theodoropoulou, Georgios Theocharis, Konstantinos Thomopoulos, Pantelis Karatzas, Konstantinos H. Katsanos, Andreas Kapsoritakis, Anastasia Kourikou, Nikoleta Mathou, Spilios Manolakopoulos, Georgios Michalopoulos, Spyridon Michopoulos, Alexandros Boubonaris, Giorgos Bamias, Vasileios Papadopoulos, George Papatheodoridis, Ioannis Papaconstantinou, Ioannis Pachiadakis, Konstantinos Soufleris, Maria Tzouvala, Christos Triantos, Eftychia Tsironi, Dimitrios K. Christodoulou, Ioannis E. Koutroubakis

**Affiliations:** 1 Dept of Gastroenterology, University Hospital Heraklion, P.O. BOX 1352, 71110 Heraklion, Crete Greece; 2 Department of Gastroenterology, Venizelio General Hospital, Heraklion, Greece; 3 Department of Gastroenterology, General Hospital of Athens “Alexandra”, Athens, Greece; 4 2nd Department of Surgery, Aretaieion Hospital, National and Kapodistrian University of Athens, Athens, Greece; 5 Department of Gastroenterology, General Hospital of Piraeus “Tzaneio”, Athens, Greece; 6 Hepato-Gastroenterology/Endoscopy Unit, 2nd Department of Internal Medicine, National and Kapodistrian University of Athens, Athens General Hospital “Heppocratio”, Athens, Greece; 7 Department of Gastroenterology, University General Hospital of Larissa, Larissa, Greece; 8 2nd Internal Medicine Department, General Hospital of Thessaloniki “Ippokratio”, Thessaloniki, Greece; 9 Gastroenterology Unit, 3rd Academic Department of Internal Medicine, National and Kapodistrian Univeristy of Athens, “Sotiria” General Hospital, Athens, Greece; 10 Department of Gastroenterology, National and Kapodistrian University of Athens, General Hospital of Athens “Laiko”, Athens, Greece; 11 Department of Gastroenterology, University General Hospital of Ioannina, Ioannina, Greece; 12 Department of Gastroenterology, “Metaxa” General Anticancer Hospital of Piraeus, Piraeus, Greece; 13 Department of Gastroenterology, General Hospital of Nikaia Piraeus “Ag. Panteleimon”-General Hospital Dytikis Attikis “Agia Varvara”, Athens, Greece; 14 Division of Gastroenterology, Department of Internal Medicine, University Hospital of Patras, Patras, Greece; 15 Department of Gastroenterology-Oncology, Theageneio Cancer Hospital of Thessaloniki, Thessaloniki, Greece; 16 Department of Gastroenterology, General Hospital of Nea Ionia “Konstantopoulio – Patision”, Athens, Greece; 17 Department of Gastroenterology, 424 General Military Hospital, Thessaloniki, Greece

**Keywords:** Anemia, Crohn’s disease, Iron, Iron deficiency, Ulcerative colitis

## Abstract

**Background:**

Anemia is a common extraintestinal manifestation of Inflammatory Bowel Disease (IBD) affecting negatively the patients’ quality of life. The aim of this study was to determine the frequency and real-life management of anemia in IBD patients in Greece.

**Methods:**

This study was conducted in 17 Greek IBD referral centers. Demographic, clinical, laboratory, IBD and anemia treatment data were collected and analyzed retrospectively.

**Results:**

A total of 1394 IBD patients [560 ulcerative colitis (UC), 834 Crohn’s disease (CD)] were enrolled. Anemia at any time was reported in 687 (49.3%) patients of whom 413 (29.6%) had episodic and 274 (19.7%) had recurrent/persistent anemia. Anemia was diagnosed before IBD in 45 (6.5%), along with IBD in 269 (39.2%) and after IBD in 373 (54.3%) patients. In the multivariate analysis the presence of extraintestinal manifestations (p = 0.0008), IBD duration (p = 0.026), IBD related surgeries and hospitalizations (p = 0.026 and p = 0.004 accordingly) were risk factors of recurrent/persistent anemia. Serum ferritin was measured in 839 (60.2%) IBD patients. Among anemic patients, 535 (77.9%) received treatment. Iron supplementation was administered in 485 (90.6%) patients, oral in 142 (29.3%) and intravenous in 393 (81%).

**Conclusions:**

The frequency of anemia in IBD patients, followed at Greek referral centers, is approximately 50%. Development of recurrent/persistent anemia may be observed in 20% of cases and is independently associated with the presence of extraintestinal manifestations, IBD duration, IBD related surgeries and hospitalizations. Anemia treatment is administered in up to $4/5$ of anemia IBD patients with the majority of them receiving iron intravenously.

## Background

Inflammatory bowel diseases (IBD) are chronic inflammatory disorders that encompass mainly Crohn’s disease (CD) and ulcerative colitis (UC). The incidence of IBD in Greece is about 10 per 100,000 inhabitants per year (6.1–8.9 for UC and 2.75–3.0 for CD) (1).

IBD patients often present extra-intestinal manifestations with anemia to be the most common (2). There is a wide range of the reported prevalence of anemia in IBD possibly reflecting the heterogeneity of the study population (in-hospital or out-patient patients), of the severity of IBD, of the IBD treatment (5-ASA, anti-TNFs etc.) as well as of the time point of the assessment (at diagnosis or during the course of IBD).

Anemia is typically classified into iron deficiency anemia (IDA), anemia of chronic disease (ACD) and anemia due to B12 or folic acid deficiency based on the ECCO guidelines. However, there is a difficulty in determining the exact type of anemia in IBD patients, since chronic blood loss, inflammation, medication toxicity, surgical procedures, malabsorption or even malnutrition can be present at the same time in an IBD patient (3, 4). Another classification of anemia that has been suggested is based on the course of anemia as episodic (if presented only once), recurrent (if Hb was restored for a period of time and then dropped below normal again) and persistent anemia (if all available values of Hb were below normal) (5). Most of the anemia’s symptoms are very common (fatigue, dizziness, dyspnea, cold skin, glossitis, hair loss) while others (impairment of cognitive function, restless legs syndrome) are less frequently seen. Anemia is of a great importance not only because of its high frequency but also because of the negative impact on work productivity and quality of life (6, 7). It is well established that anemia is positively correlated with the IBD activity, risk of surgery and prolonged hospitalization but recent studies even correlate anemia with IBD severity (5, 8).

Although anemia diagnosis in IBD is often neglected, anemia treatment should be given to all patients with low hemoglobin (Hb) particularly in the form of iron replacement, since ID is the most frequent cause of anemia in IBD. Recent data suggest that intravenous iron administration is safe, well tolerated and efficient particularly when it comes to patients with severe anemia (Hb < 10.0 g/dl), intolerant or non-responding to oral iron supplementation, and those with active disease (9).

The aims of this study were: (i) to capture the frequency of anemia in a cohort of IBD patients followed at Greek referral centers, (ii) to investigate for potential risk factors for anemia and (iii) to register real life management of anemia.

## Methods

This is a retrospective, multicenter observational study conducted in 17 IBD centers in Greece. Patients 18–80 years, with confirmed diagnosis of UC or CD for at least 3 months and having attended > 1 IBD clinic visits with available lab results permitting the evaluation of anemia were included. All patients with known comorbidities that could cause anemia independently (congestive heart failure, chronic kidney failure, active cancer, cirrhosis, Mediterranean anemia trait etc.) as well vegetarian patients were excluded. Gender, age at IBD diagnosis, disease duration at study entry for non-anemic patients and at first presentation of anemia for anemic patients, disease location and behavior for CD and disease extent for UC according to Montreal Classification, smoking habits, extra-intestinal manifestations, IBD-related hospitalizations and surgeries (right hemicolectomy, enterectomy, colectomy), IBD treatment and C-reactive protein (CRP) at entry for non-anemic and at first presentation of anemia for anemic patients were recorded in a pre-defined standardized report form. Clinical disease activity was evaluated with Harvey- Bradshaw score (HBI) and Simple Clinical Colitis Score (SCCAI) for CD and UC respectively and Short Inflammatory Bowel Disease Questionnaire (SIBDQ) was used to evaluate quality of life, at the study entry. Data was extracted retrospectively from the local IBD databases of 17 Greek centers for a period of 12 months (from February 2019 until January 2020). There has not been adopted a special policy of checking either the hemoglobin or the general blood count, specifically for this study protocol and the frequency of the lab tests was according to the clinical practice (usually every 3 months for those receiving biologics, immunomodulators or combination treatment and every 6 months for those on exclusively non-immunosuppressive treatment).

Anemia was defined according to World Health Organization (WHO) criteria when hemoglobin (Hb) was below 12.0 g/dl for non-pregnant women and below 13.0 g/dl for men (7). Anemia was characterized as episodic, if presented only once, recurrent, if Hb was restored for a period of time and then dropped below normal again, and persistent if all available values of Hb were below normal. Treatment of anemia was also captured. Hemopoietic response was also evaluated, in patients treated with iron, where there were available laboratory data. Responders to the treatment of anemia were considered those with an increase of Hb by > 2 g/dl or when Hb returned to normal levels within 4 weeks after iron administration (11, 12).

### Statistical analysis

Data are presented either as mean (± SD) for normally distributed variables or as median (IQR, range) for non-normally distributed. Categorical data were analyzed with the chi-square or Fischer’s exact test. Independent factors associated with persistent/recurrent anemia were assessed by multivariate analysis based on binary logistic regression analysis and including those covariates with p < 0.10 in the univariate analysis. Odds ratios (OR) were calculated with 95% confidence intervals (CI). Tests were two-sided and P values < 0.05 were considered to be statistically significant. The statistical program used was MedCalc (MedCalc Software Ltd, Belgium).

## Results

A total of 1394 patients met the inclusion criteria and formed the study population. Of those, 560 patients with UC and 834 with CD were included. Demographics and clinical characteristics of the population under investigation are shown in Table 1.

**Table 1:** Demographics and clinical characteristics of study population (N = 1394)

	Total IBD	CD	UC
Number (%)	1394 (100)	834 (59.8)	560 (40.2)
Median diagnosis age (years, range)	33 (23–46)	32 (22–46)	35 (24–46)
Sex (females, %)	635 (45.6)	389 (46.6)	246 (43.9)
Median IBD duration^a^ (years, range)	3 (1–9)	3 (1–8.25)	3 (1–10)
Active smokers (%)	442 (31.7)	330 (39.5)	112 (20)
Ex-smokers (%)	366 (26.2)	189 (22.7)	177 (31.6)
Never smoked (%)	586 (42.1)	315 (37.8)	271 (48.4)
Median BMI (range)	24.5 (21.5–27.8)	24.3 (21.5–27.9)	24.7 (21.6–27.7)
Montreal classification for UC			
Proctitis (E1, %)			63 (11.25)
Left sided colitis (E2, %)			237 (42.5)
Extensive colitis (E3, %)			259 (46.25)
Montreal classification for CD			
I nflammatory (B1, %)		515 (61.8)	
Stricturing (B2, %)		190 (22.8)	
Penetrating (B3, %)		129 (15.4)	
Perianal (p, %)		165 (19.8)	
Ileum (L1, N, %)		376 (45.0)	
Colon (L2, N, %)		79 (9.5)	
Ileocolonic (L3, N, %)		380 (45.5)	
Upper GI (L4, N, %)		114 (13.7)	
EIMs (%)	469 (33.6)	325 (38.9)	144 (25.7)
Anti-TNFs (N, %)	692 (49.6)	495 (59.4)	197 (35.2)
Immunomodulators (N, %)	529 (37.9)	380 (45.6)	149 (26.6)
Other biologics (N, %)	230 (16.5)	131 (15.7)	99 (17.7)
IBD-related surgery (N, %)	176 (12.6)	156 (18.7)	20 (3.6)

UC, Ulcerative Colitis; CD, Crohn’s Disease; BMI, Body Mass Index; IBD, Inflammatory Bowel Disease; GI, Gastrointestinal; EIMs, Exta-intestinal Manifestations; Anti-TNFs, Anti-Tumor Necrosis Factors

^a^At first presentation of anemia for anemic patients or at entry for non-anemic patients

The median age at diagnosis was 32 (IQR 24, 22–46) years for CD and 35 (IQR 22, 24–46) years for UC. Females in CD were 389 (46.6%) whereas in UC were 246 (43.9%). Active smokers were more in CD (39.5%) than in UC (20%) (p < 0.0001). Extra-intestinal manifestations, other than anemia, were present in 325 (38.9%) CD and 144 (25.7%) UC patients (p < 0.0001). A history of major IBD-related surgery was reported by 176 (12.6%) patients, 156 (18.7%) with CD and 20 (3.6%) with UC (p < 0.0001). Anti-TNFs exposed were more in CD (59.4%) than in UC (35.2%) and use of immunomodulators (Azathioprine, Methotrexate, 6-Mercaptopurine) was also more frequent in CD than in UC patients (45.6% vs 26.6%) (both with p < 0.0001). The patients on newer biologics (Vedolizumab and Ustekinumab) were 230 (151 and 79 accordingly) are referred together as “other biologics” on the Tables (Table 1, 2, 4). Regarding Tofacitinib, at that timepoint when the study recruited patients, was not available in Greece.

**Table 2:** Patients’ characteristics according to their anemia history (N = 1394)

	Group A No anemia	Group B Episodic anemia	Group C Recurrent/persistent anemia	P
Number (%)	707 (50.7)	413 (29.6)	274 (19.7)	
Males/females (%)	432/275 (61.1/38.9)	208/205 (50.4/49.6)	119/155 (43.4/56.6)	< 0.0001
CD/UC (%)	412/ 295 (58.3/41.7)	236/177 (57.1/42.9)	186/88 (67.9/32.1)	0.0089
Median IBD duration^a^ (years, range)	5 (2–11)	1 (0–4)	2 (0–8)	< 0.0001
Median diagnosis age (years, range)	35 (24–46)	32 (23–45.5)	30 (21–46)	< 0.0001
Active smokers (%)	238 (33.7)	121 (29.3)	83 (30.3)	0.2356
Ex-smokers (%)	205 (29)	96 (23.2)	64 (23.3)	0.0435
Never smoked (%)	264 (37.3)	195 (47.2)	127 (46.4)	0.0008
Median BMI (range)	25.2 (22.5–28.6)	24.2 (21.3–27.25)	23.5 (20.7–27.0)	< 0.0001
*Montreal classification for UC*				
Proctitis (E1, %)	53 (18)	7 (3.9)	3 (3.4)	< 0.0001
Left sided colitis (E2, %)	129 (43.7)	75 (42.4)	33 (37.5)	0.5834
Extensive colitis (E3, %)	113 (38.3)	95 (53.7)	52 (59.1)	0.0002
*Montreal classification for CD*				
Inflammatory (B1, %)	272 (66)	154 (65.2)	89 (47.8))	< 0.0001
Stricturing (B2, %)	84 (20.4)	45 (19.1)	61 (32.8)	0.6111
Penetrating (B3, %)	56 (13.6)	36 (15.2)	36 (19.4)	0.6050
Perianal (p, %)	80 (19.4)	42 (17.8)	42 (22.5)	0.4901
Ileum (L1, N, %)	209 (50.7)	97 (41.1)	70 (37.6)	0.0037
Colon (L2, N, %)	35 (8.5)	23 (9.7)	20 (10.8)	0.6575
Ileocolonic (L3, N, %)	168 (40.8)	116 (49.2)	96 (51.6)	0.0191
Upper GI (L4, N, %)	46 (11.2)	41 (17.4)	27 (14.5)	0.0766
Disease activity—clinically judged	176 (24.9)	238 (57.6)	145 (52.9)	< 0.0001
EIMs (%)	199 (28.1)	138 (33.4)	131 (47.8)	< 0.0001
Anti-TNFs (N, %)	261 (36.9)	242 (58.6)	188 (68.6)	< 0.0001
Immunomodulators (N, %)	225 (31.8)	175 (42.4)	129 (47.1)	< 0.0001
Other biologics (N, %)	82 (11.6)	90 (21.8)	58 (21.2)	0.0624
IBD-related surgery (N, %)	61 (8.6)	51 (12.3)	64 (23.4)	< 0.0001

Group A never anemic patients, Group B episodically anemic patients, Group C recurrently/persistently anemic patients

UC, Ulcerative Colitis; CD, Crohn’s Disease; BMI, Body Mass Index; IBD, Inflammatory Bowel Disease; GI, Gastrointestinal; EIMs, Exta-intestinal Manifestations; Anti-TNFs, Anti-Tumor Necrosis Factors

^a^At first presentation of anemia for anemic patients or at entry for non-anemic patients

**Fig. 1:**
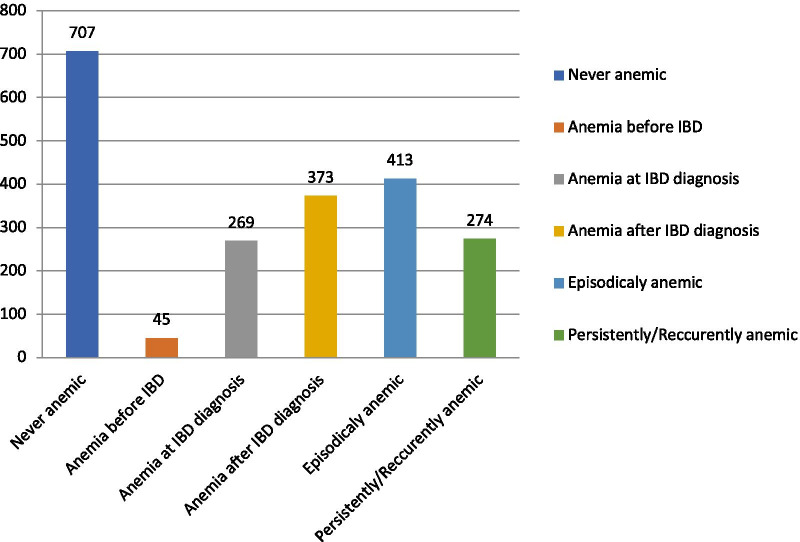
Time point and status of Anemia in IBD patients (N = 1394)

In total, 687/1394 (49.3%) had a diagnosis of anemia. Anemia was initially detected more than one year before IBD in 45 patients (6.5%, 31 CD and 14 UC), concurrently with IBD or less than 1 year before in 269 (39.2%, 156 CD and 113 UC) and after IBD diagnosis in 373 (54.3%, 236 CD and 137 UC) (Fig. 1). The study population was divided into three groups: non-anemic patients at any time (group A), episodically anemic (group B), and recurrently or persistently anemic patients (group C) (Table 2). There were more males than females in group A (61.1% vs 38.9%, p < 0.0001) whereas there was no significant difference in the other two groups regarding gender. CD was the more frequent diagnosis in groups B and C (p = 0.0039 and p = 0.0043).

Median age at diagnosis, median disease duration and median BMI were higher in group A compared to the other two groups (all with p < 0.0001, Table 2). UC proctitis was more common and extensive colitis less common in group A (p < 0.0001 and p = 0.0002 respectively). In CD, patients non-stricturing non-penetrating phenotype (B1) was more common in group A (p < 0.0001) as well as ileal (L1) disease (p = 0.0037) whereas ileocolonic disease (L3) was more common in group C (p = 0.0191, Table 2).

Clinically judged active disease was more frequently seen in groups B and C (p < 0.0001). Median CRP levels as well as median HBI (for CD) and SCCAI (for UC) were significantly higher in groups B and C (Table 3). Quality of life as measured with SIBDQ was worse in groups B and C (p < 0.0001). Furthermore, patients in these groups were more often under treatment with anti-TNFs or immunomodulators (p < 0.0001). IBD-related surgeries were also more often among anemic patients (p < 0.0001).

**Table 3:** Disease activity in IBD anemic (Group B and C) and non-anemic patients (Group A)

Characteristics	Anemic patients Group B and C (N = 687)	Non-anemic patients Group A (N = 707)	p
Disease activity (clinically judged)^a^ (%)	382 (55.6%)	176 (24.9%)	< 0.0001
Median CRP^a^ mg/dl	2.00 (0.50–7.00)	0.40 (0.14–1.3)	< 0.0001
Median SIBDQ^a^	50 (39–60)	61 (51–67)	< 0.0001
Median HBI^a^	5 (3–7)	3 (1–4)	< 0.0001
Median SCCAI^a^	5 (3–8)	1 (0–4)	< 0.0001
Median Ferritin^a^ ng/ml	23.03 (10–65.9)	70 (39–113.2)	< 0.0001

Group A never anemic patients, Group B episodically anemic patients, Group C recurrently/persistently anemic patients

CRP, C-Reactive Protein; SIBDQ, Simple Inflammatory Bowel Disease *Questionnaire*; HBI, Harvey-Bradshaw Index; SCCAI, Simple Colitis Activity Index

^a^At entry

In the univariate analysis, persistent/recurrent anemia was found to be associated with CD diagnosis [OR 1.59 (1.16–2.19), p = 0.0044], median disease duration [1.03 (1.01–1.05), p = 0.0100], penetrating or stricturing CD [2.07 (1.40–3.07), p = 0.0003], presence of EIMs [1.82 (1.33–2.49), p = 0.0002] use of anti-TNF [1.53 (1.11–2.12), p = 0.0090], IBD related hospitalizations [2.39 (1.56–3.66), p = 0.0001] and IBD related surgeries [2.15 (1.43–3.23), p = 0.0002] (Table 4). In the multivariate analysis, parameters that remained significantly associated with persistent/recurrent anemia were median disease duration [1.03 (1.00–1.05), p = 0.0259], presence of EIMs [1.79 (1.27–2.52), p = 0.0008], IBD related hospitalizations [1.96 (1.25–3.09), p = 0.0035], and IBD related surgeries [1.73 (1.07–2.78), p = 0.0257] (Table 4).

**Table 4:** Risk factors for persistent/recurrent anemia (group C)

Risk factors	Univariate analysis	P	Multivariate analysis	P
	OR (95% CI)		OR (95% Cl)	
Female sex	1.32 (0.97–1.79)	0.0804	0.78 (0.56–1.10)	0.1555
CD diagnosis	1.59 (1.16–2.19)	0.0044	0.77 (0.54–1.10)	0.1519
Median IBD duration	1.0304 (1.01–1.05)	0.0100	1.03 (1.00–1.05)	0.0259
Median diagnosis age	0.9971 (0.99–1.01)	0.5563		
Active smokers	1.04 (0.75–1.45)	0.8113		
CRP > 3 mg/dl	1.11 (0.79–1.56)	0.5512		
Extensive colitis	1.28 (0.76–2.14)	0.3568		
Penetrating or stricturing CD	2.07 (1.40–3.07)	0.0003		
Ileocolonic	1.09 (0.74–1.61)	0.6464		
EIMs	1.82 (1.33–2.49)	0.0002	1.79 (1.27–2.52)	0.0008
Major disease surgeries	2.15 (1.43–3.23)	0.0002	1.73 (1.07–2.78)	0.0257
IBD hospitalizations^a^	2.39 (1.56–3.66)	0.0001	1.96 (1.25–3.09)	0.0035
Immmunomodulators	1.20 (0.89–1.64)	0.2346		
Anti-TNF any time	1.53 (1.11–2.12)	0.0090	0.79 (0.55–1.14)	0.2062
Other biologics at any time	1.48 (0.82–2.68)	0.1944		

CD, Crohn’s Disease; IBD, Inflammatory Bowel Disease; CRP, C-Reactive Protein; EIMs, Exta-intestinal Manifestations; Anti-TNFs, Anti-Tumor Necrosis Factors

^a^IBD hospitalizations n = 5 the last 5 years

Anemia and iron status were mainly assessed with Hb and ferritin measurement. Ferritin value was available in 839/1394 patients (60.2%) and the median value was 43 ng/ml (IQR 76.7, 13–89.7). Moreover, ferritin was measured in 523 of the 687 (76.1%) anemic patients [median value 23 ng/ml (IQR 55.9, 10–65.9)]. Ferritin below 30 ng/ml was detected in 377 patients (72%) [median value 8 ng/ml (IQR 8, 7–17)] whereas ferritin above 30 ng/ml and below 100 ng/ml with CRP above 1 mg/dl was measured in 61 patients (11.7%) [median value 55.2 ng/ml (IQR 28.5, 41–69.5)] which is, according to the ECCO guidelines, compatible with Iron Deficiency Anemia (IDA). Furthermore, anemia of chronic disease (ferritin > 100 ng/ml) was detected in 85 patients (16.3%) [median value 176 ng/ml (IQR 118, 134.8–252.8)].

From the 687 anemic IBD patients, 535 (77.9%, 327 CD, 208 UC) received treatment for their anemia. The vast majority (485/535, 90.6%) were treated with iron supplementation either as an oral in 142 (29.3%) or as an intravenous formulation in 393 (81%) with a small percentage of patients having received both successively. Of those received iron intravenously, 351 (89.3%) responded, whereas 77 (54.2%) responded of those received per os iron. Thirty one out of the 142 patients (21.8%) that received iron orally presented with adverse events (mostly gastrointestinal symptoms such as abdominal pain and constipation) that eventually led to cessation of the treatment. On the other hand, 31/393 patients (7.9%) that iron was given intravenously experienced adverse events during infusion (allergic reactions, skin rash) and 11/393 (2.8%) post infusion (headache, dizziness, hypophosphatemia). B12 vitamin was administered in 113 (21.1%) and folic acid in 182 (33.9%) patients based on laboratory findings compatible with deficiency. Sixty-eight patients (12.7%) needed blood transfusion. More details for the treatment of anemia are shown in Table 5.

**Table 5:** Treatment of anemic IBD patients (N = 687)

	Anemia total (N = 687)	Episodic anemia Group B (N = 413)		Recurrent/Persistent anemia Group C (N = 274)	P
Anemia treatment (%)	535 (77.9)		300 (72.6)	235 (85.8)	< 0.0001
CD/UC (%)	327/208 (61.2/38.8)		167/133 (55.7/44.3)	160/75 (68.1/31.9)	
Iron Administration (%)	485 (90.6)		286 (95. 3)	213 (90.6)	
Iron PO/IV^a^ (%)	142/392 (29.3/ 81)		72/214 (25.2/75)	70/178 (32.9/83.6)	
B12 Administration (%)	113 (21.1)		48 (16)	65 (27.6)	0.0014
Folic acid Administration^b^ (%)	182 (33.9)		80 (26.7)	102 (43.4)	< 0.0001
Blood Transfusion (%)	68 (12.7)		36 (12)	32 (13.6)	0.0061

Group A never anemic patients, Group B episodically anemic patients, Group C recurrently/persistently anemic patients

UC, Ulcerative Colitis; CD, Crohn’s Disease; PO, Per Os; IV, Intravenously

^a^Patients who were initially treated with oral iron, were subsequently treated with intravenous iron due to either lack of response or adverse events that led to discontinuation of the oral formulation

^b^Apart from concurrent administration with MTX

## Discussion

The present study showed that the frequency of anemia in Greek IBD patients followed at tertiary referral centers is 49.3% meaning that almost half of those patients experience anemia, which is similar to previous reports from other countries (2, 13–16). Anemia was present more frequently in CD patients and in females which is also in accordance to other studies (13, 15, 17). It is of notice that there were few patients (6.5%) with anemia diagnosed more than one year before IBD diagnosis, whereas anemia was diagnosed at IBD diagnosis or less than one year before in 39.2% of the patients. In newly diagnosed IBD patients of a population‐based inception cohort the prevalence of anemia has been reported to be 48.8% for CD and 20.2% for UC (18). Data on the prevalence of anemia before IBD diagnosis is limited (19).

Anemia was found to be episodic (presented once) in 29.6% and persistent/recurrent in 19.7% of the patients. Recurrent/persistent is considered the most serious type of anemia in IBD since it has been associated with severe and disabling disease (5). In our study recurrent/persistent anemia was found to be significantly and independently associated with disease duration, presence of EIMs, history of major IBD related surgeries and IBD related hospitalizations (n = 5 the last 5 years). It is of notice that EIMs (other than anemia) were found to be strongly correlated with the development of persistent/recurrent anemia (p = 0.0008). Even though anemia itself is one of the most common EIM, it seems that the presence of EIMs (arthritic, skin etc.) could independently be a causative factor for anemia of chronic disease, which is a possible explanation for the above-mentioned strong association between anemia and EIMs. On the other hand, both anemia and EIMs have been reported to be associated with worse disease outcome and severe disease (20).

There is a known association between IBD activity and the presence of anemia (21) something that is in accordance with our results since anemic patients had clinically judged active IBD more often than non-anemic patients. Moreover, median CRP as well as median HBI (for CD) and median SCAAI (for UC) were higher and median IBDQ was lower in patients with anemia. The IBD activity either expressed by clinical judgment or by clinical scores (HBI, SCAAI), accompanied by patients’ reported outcomes (IBDQ) reflects a snapshot of a particular moment during disease course. On the other hand, the IBD severity is a wider term incorporating the overall disabling course of the disease with worst outcome, including surgeries frequent hospitalizations etc. Recent reports (5) and the present study support that persistent or recurrent anemia correlates with more aggressive or disabling disease in patients with IBD.

Anemia in IBD patients is considered to be often neglected during the course and treatment of the primary disease, since according to literature review 68.6% of the anemic UC patients in USA were not further investigated (with ferritin etc.) and even 25% of IDA IBD patients remained untreated (22). Furthermore, in Switzerland anemia treatment (iron, B12 administration etc.) occurred only in 40% of patients in private practice and 43% in University Hospitals (23). On the other side, this study showed that the majority of the anemic IBD patients (77.9%) received proper treatment. In this context, most of the patients (90.6%) received iron supplementation with 81% of them receiving iron intravenously. This could be partially attributed to the fact that our study was conducted in referral centers and may not be representative of the real practice in the general IBD population. Our findings are in contrast with earlier studies in Germany (24) and other European countries, in which most of the IBD anemic patients (92%) are treated with iron, but in the vast majority (67%) with oral and only 28% with intravenous formulations (25). An exception to this strategy constitutes Sweden and Switzerland, where iron supplementation was intravenously administered in 72% and 52% accordingly. In the present study ferritin was measured in 60.2% of the study population and in 76.1% of the anemic patients. This percentage is higher than what was previously referred in the literature (22) and even better than what is recorded in pediatric IBD patients. Although a more meticulous approach is expected in pediatric patients, Miller et al. mentioned ferritin measurement only 1 out of 5 anemic pediatric IBD patients (26).

Currently there are no pre-existing data for the frequency, diagnosis and treatment of anemia in Greek IBD patients. Anemia and iron status are mainly assessed with Hb and ferritin since measurement of transferrin saturation (TSAT) or other blood markers of anemia are not widely available in the everyday clinical practice in Greece. On the contrary TSAT was available in 61% of the anemic patients in the study of Blumenstein et al. in Germany (24) whereas in other studies this percentage reached 25% (3). Our findings demonstrate that intravenous iron is implemented more frequently compared to oral administration in IBD patients in Greece. This is in contrast with previous European studies something probably attributed to the fact that these were conducted some years ago and before the development of recent guidelines.

According to ECCO guidelines screening for anemia should be implemented every 6 to 12 months for patients in remission or mild disease and at least every 3 months in those with active disease. Furthermore, a ferritin threshold of 30 ng/ml for patients with inactive disease and of 100 ng/ml for patients with active IBD with raised inflammation markers such as CRP has been proposed for the diagnosis of IDA. Moreover, in the presence of inflammation, the diagnostic criteria for ACD are serum ferritin > 100 ng/ml and TSAT < 20%. If serum ferritin level is between 30 and 100 ng/ml, a combination of true iron deficiency and ACD is more probable (11).

This study has several strengths but some limitations as well. First, this is a retrospective study, conducted in 17 different referral centers, where more severe IBD cases are followed up and further not all of them are abide by the anemia guidelines with the same way. Second, selection bias may exist because patients visit these centers and received treatment for anemia more frequently. What is more, the IBD activity assessment was made based on clinical scores (HBI, SCAAI) since the gold-standard, which is endoscopy, could not be applied in a retrospective study in 17 different IBD centers and furthermore fecal calprotectin is not yet widely available in Greece and the national health system does not reimburse IBD patients for this laboratory examination. On the other hand, this study included a large number of IBD patients and constitutes the first attempt to record anemia burden in Greek IBD patients and relevant treatment strategy. Moreover, a different classification of anemia based on the course of anemia (episodic, persistent and recurrent) together with the classic one (iron deficiency anemia, anemia of chronic disease and B12 or folic acid deficiency) were applied in order to depict more accurately the overall burden of anemia in IBD patients. Last, this study aimed to raise awareness towards anemia diagnosis and treatment that seems to be often neglected.

In conclusion, half of the Greek IBD patients present anemia during their disease course. One out of five patients present recurrent/persistent anemia despite the treatment for their primary disease. Risks factors for recurrent/persistent anemia are the presence of extraintestinal manifestations, IBD duration, IBD related surgeries and hospitalizations. In real life in referral Greek IBD centers, 77.9% of anemic patients receive anemia treatment with the majority of them receiving iron intravenously.

## Conclusions

The frequency of anemia in IBD patients followed at Greek referral centers is approximately 50%. Development of recurrent/persistent anemia may be observed in 20% of cases and is independently associated with presence of extraintestinal manifestations, IBD duration and IBD related surgeries and hospitalizations. Anemia treatment is administered in up to $4/5$ of anemia IBD patients with the majority of them receiving iron intravenously.

## Data Availability

Data are available on request. *Data underlying this article will be shared on reasonable request to the corresponding author.*

## Abbreviations

IBD: Inflammatory bowel disease
UC: Ulcerative colitis
CD: Crohn’s disease
IBDU: Inflammatory bowel disease unclassified
5-ASA: 5-Sibdqaminosalicylic acid
ID: Iron deficiency
Hb: Hemoglobin
CRP: C-reactive protein
HBI: Harvey-Bradshaw score
SCCAI: Simple clinical colitis score
SIBDQ: Short inflammatory bowel disease questionnaire
WHO: World health organization
SD: Standard deviation
IQR: Interquartile range
CI: Confidence interval
BMI: Body mass index
EIMs: Extraintesinal manifestations
IDA: Iron deficiency anemia
TSAT: Transferin saturation
ACD: Anemia of chronic disease
ECCO: European Chron’s and colitis organization
