# Correction to: The burden and management of anemia in Greek patients with inflammatory bowel disease: a retrospective, multicenter, observational study

**DOI:** 10.1186/s12876-021-01872-9

**Published:** 2021-07-29

**Authors:** Kalliopi Foteinogiannopoulou, Konstantinos Karmiris, Georgios Axiaris, Magdalini Velegraki, Antonios Gklavas, Christina Kapizioni, Charalabos Karageorgos, Christina Kateri, Anastasia Katsoula, Georgios Kokkotis, Evgenia Koureta, Charikleia Lamouri, Panagiotis Markopoulos, Maria Palatianou, Ploutarchos Pastras, Konstantinos Fasoulas, Olga Giouleme, Evanthia Zampeli, Aggeliki Theodoropoulou, Georgios Theocharis, Konstantinos Thomopoulos, Pantelis Karatzas, Konstantinos H. Katsanos, Andreas Kapsoritakis, Anastasia Kourikou, Nikoleta Mathou, Spilios Manolakopoulos, Georgios Michalopoulos, Spyridon Michopoulos, Alexandros Boubonaris, Giorgos Bamias, Vasileios Papadopoulos, George Papatheodoridis, Ioannis Papaconstantinou, Ioannis Pachiadakis, Konstantinos Soufleris, Maria Tzouvala, Christos Triantos, Eftychia Tsironi, Dimitrios K. Christodoulou, Ioannis E. Koutroubakis, the Hellenic group for the study of IBD, Kalliopi Foteinogiannopoulou, Konstantinos Karmiris, Georgios Axiaris, Magdalini Velegraki, Antonios Gklavas, Christina Kapizioni, Charalabos Karageorgos, Christina Kateri, Anastasia Katsoula, Georgios Kokkotis, Evgenia Koureta, Charikleia Lamouri, Panagiotis Markopoulos, Maria Palatianou, Ploutarchos Pastras, Konstantinos Fasoulas, Olga Giouleme, Evanthia Zampeli, Aggeliki Theodoropoulou, Georgios Theocharis, Konstantinos Thomopoulos, Pantelis Karatzas, Konstantinos H. Katsanos, Andreas Kapsoritakis, Anastasia Kourikou, Nikoleta Mathou, Spilios Manolakopoulos, Georgios Michalopoulos, Spyridon Michopoulos, Alexandros Boubonaris, Giorgos Bamias, Vasileios Papadopoulos, George Papatheodoridis, Ioannis Papaconstantinou, Ioannis Pachiadakis, Konstantinos Soufleris, Maria Tzouvala, Christos Triantos, Eftychia Tsironi, Dimitrios K. Christodoulou, Ioannis E. Koutroubakis

**Affiliations:** 1 Gastroenterology Department, University Hospital of Heraklion, Medical School University of Crete, P.O. BOX 1352, 71110 Heraklion, Crete Greece; 2 Department of Gastroenterology, Venizelio General Hospital, Heraklion, Greece; 3 Department of Gastroenterology, General Hospital of Athens “Alexandra”, Athens, Greece; 4 2nd Department of Surgery, Aretaieion Hospital, National and Kapodistrian University of Athens, Athens, Greece; 5 Department of Gastroenterology, General Hospital of Piraeus “Tzaneio”, Athens, Greece; 6 Hepato-Gastroenterology/Endoscopy Unit, 2nd Department of Internal Medicine, National and Kapodistrian University of Athens, Athens General Hospital “Heppocratio”, Athens, Greece; 7 Department of Gastroenterology, University General Hospital of Larissa, Larissa, Greece; 8 2nd Internal Medicine Department, General Hospital of Thessaloniki “Ippokratio”, Thessaloniki, Greece; 9 Gastroenterology Unit, 3rd Academic Department of Internal Medicine, National and Kapodistrian Univeristy of Athens, “Sotiria” General Hospital, Athens, Greece; 10 Department of Gastroenterology, National and Kapodistrian University of Athens, General Hospital of Athens “Laiko”, Athens, Greece; 11 Department of Gastroenterology, University General Hospital of Ioannina, Ioannina, Greece; 12 Department of Gastroenterology, “Metaxa” General Anticancer Hospital of Piraeus, Piraeus, Greece; 13 Department of Gastroenterology, General Hospital of Nikaia Piraeus “Ag. Panteleimon”-General Hospital Dytikis Attikis “Agia Varvara”, Athens, Greece; 14 Division of Gastroenterology, Department of Internal Medicine, University Hospital of Patras, Patras, Greece; 15 Department of Gastroenterology-Oncology, Theageneio Cancer Hospital of Thessaloniki, Thessaloniki, Greece; 16 Department of Gastroenterology, General Hospital of Nea Ionia “Konstantopoulio – Patision”, Athens, Greece; 17 Department of Gastroenterology, 424 General Military Hospital, Thessaloniki, Greece

## Correction to: BMC Gastroenterol 21, 269 (2021). https://doi.org/10.1186/s12876-021-01826-1

Following publication of the original article [1], it was reported that the affiliation of the corresponding author should be changed from “Dept. of Gastroenterology, University Hospital Heraklion, Heraklion, Crete, Greece" to "Gastroenterology Department, University Hospital of Heraklion, Medical School University of Crete, Greece''.

The original article [1] has been corrected.
